# Gold Blindness, or Retinal Asthenopia

**Published:** 1901-06-15

**Authors:** 


### GOLD BLINDNESS, OR RETINAL ASTHENOPIA.

This is defective or obscure vision resulting from long concentration of the vision in gold filling or in alloying or refining gold by the dentist, and is due to over-stimulating action of the yellow rays on the rods and cones of the retina. It may be prevented or overcome by wearing properly tinted eye glasses. Slightly tinted arundel or violet colors are effective. Dr. L. Webster Fox, of Philadelphia, in the *Dental Cosmos*, page 121, 1901, says that general health impairment may be a promoting cause. The use of tobacco, alcohol and improper foods should be discarded. Meats and other foods which tend to produce uric acid should be avoided. Drink pure water, take long walks and indulge in healthful outdoor sports, such as golf. Decorate the walls of the operating room with pictures of green fields, country lanes, trees, etc. If practicable, have a growing lawn to look out upon when the eyes are tired from over-concentration. Keep the head erect and change position as often as possible. Avoid tight neckwear which impedes return blood flow and causes excessive blood pressure on the capillaries. Avoid reading lying down or when mentally and physically exhausted. Have the eyes examined to avoid errors of refraction.

